# Impact of Toxic Cyanobacterial Blooms on Eurasian Perch (*Perca fluviatilis*): Experimental Study and *In Situ* Observations in a Peri-Alpine Lake

**DOI:** 10.1371/journal.pone.0052243

**Published:** 2012-12-18

**Authors:** Benoît Sotton, Jean Guillard, Sylvie Bony, Alain Devaux, Isabelle Domaizon, Nicolas Givaudan, François Crespeau, Hélène Huet, Orlane Anneville

**Affiliations:** 1 INRA, UMR CARRTEL, Thonon Les Bains, France; 2 Université de Lyon, LEHNA, UMR 5023, ENTPE, Vaulx en Velin, France; 3 UMR CNRS Ecobio 6553, Université de Rennes 1, Campus de Beaulieu, Rennes, France; 4 Laboratoire d'Anatomie Pathologique, Ecole Nationale Vétérinaire d'Alfort, Maisons-Alfort, France; 5 INRA, USC IGH, ENTPE, Vaulx en Velin, France; Federal University of Rio de Janeiro, Brazil

## Abstract

Due to the importance of young-of-the-year (YOY) perch in the peri-alpine regions where they are consumed, the microcystin (MC) contamination of YOY perch was analysed both in field (Lake Bourget, France) and experimentally using force-feeding protocols with pure MCs. *In-situ*, schools of YOY perch present in the epilimnion of the lake were never found in direct contact with the *P. rubescens* blooms that were present in the metalimnion. However, MCs were detected in the muscles and liver of the fish and were thus assumed to reach YOY perch through dietary routes, particularly *via* the consumption of MC-containing *Daphnia*. Force-feeding experiment demonstrates the existence of MC detoxification/excretion processes and suggests that *in situ*, YOY perch could partly detoxify and excrete ingested MCs, thereby limiting the potential negative effects on perch populations under bloom conditions. However, because of chronic exposure these processes could not allow for the complete elimination of MCs. In both experimental and *in situ* studies, no histological change was observed in YOY perch, indicating that MC concentrations that occurred in Lake Bourget in 2009 were too low to cause histological damage prone to induce mortality. However, Deoxyribonucleic acid (DNA) damages were observed for both the high and low experimental MC doses, suggesting that similar effects could occur *in situ* and potentially result in perch population disturbance during cyanobacterial blooms. Our results indicate the presence of MCs in wild perch, the consumption of this species coming from Lake Bourget is not contested but more analyses are needed to quantify the risk.

## Introduction

During the past century, toxic cyanobacterial blooms have appeared worldwide as a result of the eutrophication of freshwater ecosystems. Toxic cyanobacteria produce a wide variety of metabolic compounds that are toxic for many aquatic organisms and humans [1]–[3]. In the aquatic sciences, the most studied toxic compounds produced by cyanobacteria are the microcystins (MCs), of which more than 80 variants have been characterised [4]–[5]. MCs are known to inhibit protein phosphatases 1 and 2A (PP1 and PP2A) by, an initial non-covalent and reversible binding followed by a final covalent and irreversible linkage step [6] and thereby cause oxidative stress, apoptosis and the disruption of many cellular functions. MCs may act as tumour promoters and as genotoxicants, as shown recently in whitefish [7]. Deleterious effects of MCs on the physiology and behaviour of various fish species have been observed [8], [9]. Histological disturbances, particularly in the liver, kidney, gills and intestine, have been observed in fish exposed to cyanobacteria and their toxins [10]–[12].

A large array of xenobiotics is known to be metabolised by fish detoxification systems [13]. The detoxification process involves a variety of enzymes that are implicated in the oxidation, reduction, hydrolysis and conjugation of xenobiotics present in organisms. The most studied detoxification enzymes in fish are the glutathione S-transferases (GSTs) that are responsible for the conjugation of MCs with glutathione (GSH) prior to biliary excretion [7], [14]. Experimental studies on the detoxification of MCs in various fish species have shown that this “natural defence” is species-specific and that the concentrations of MCs in fish are dose dependent [15]. Fish chronically assimilate MCs by the ingestion of cyanobacteria and contaminated zooplankton and the flow of water through their gills, leading generally to the gradual accumulation of toxins in organs [16]. However, exposure to MCs may or may not result in MC accumulation depending on the dynamics and intensity of the MCs intake and detoxification process. The measurement of MC levels in fish sampled *in situ* is essential, and the interpretation of MC concentration requires a good understanding of the dynamics of detoxification for the particular fish species being studied. The histological and genetic impacts of low toxin concentrations are difficult to evaluate in the field, primarily because of the lack of a reference state. Experimental data are therefore needed to facilitate the interpretation of field observations and to accurately assess the potential impact of MCs on fish species. For these reasons, we combined field and experimental approaches to better estimate the transfer of toxins to natural fish populations and to evaluate the potential impact of toxins on individual fish. We studied perch (*Perca fluviatilis*), a target fish species in recreational and commercial fisheries in peri-alpine lakes that has been commercialised in both its adult and young-of-the-year (YOY) stages.

In Lake Bourget (France), toxic blooms of the cyanobacterium *Planktothrix rubescens* occur [17] and produce primarily two MC variants termed MC-LR and MC-RR [18]–[20]. We used an ecosystemic approach to study *in situ* the exposure of a YOY perch population to *P. rubescens* by (i) identifying the main routes of fish exposure to MCs, (ii) assessing toxin accumulation in the fish during chronic exposure to a cyanobacterial bloom and (iii) evaluating the subsequent histological effects in the fish. Parallel experimental studies using environmentally relevant doses of MC-LR were performed in the laboratory to (i) characterise the temporal dynamics of intoxication and detoxification and (ii) assess the histological and genotoxic impacts in YOY perch exposed to environmentally relevant doses of MC-LR.

## Materials and Methods

### Ethical statement

Investigations were conducted according to the international guiding principles for the use and care of laboratory animals, and in compliance with French regulations (N° B 74 300-4) on animal welfare approved by the French Ministry of Ecology and Sustainable Development. This study was approved by the ethic committee of the Departmental Direction of Veterinary Services of Haute-Savoie (DDSV). *In-situ* sampling was made on public area with a sampling authorisation (N° 2012–209) delivered by the Prefecture of Savoie and did not involve protected species.

*In-situ* fish were captured by pelagic trawl and quickly killed, as experimental fish, by a cranial choc.

### Study site

Lake Bourget (45°44′N, 5°52′E, 231 m altitude) is a mesotrophic, warm, monomictic lake located in eastern France. It has an area of 42 km^2^, a total volume of 3.5×10^9^ m^3^ and maximum and average depths of 145 and 80 m, respectively [19]. Its water is used as a source of drinking water and for recreational activities and fisheries. The cyanobacterium *P. rubescens* was first reported in the lake in the 1950 s [21]. The occurrence and intensity of its blooms have been precisely studied since the 1990 s [17]. *P. rubescens* is present in the metalimnion of the lake during the summer [22].

### *In situ* sampling

Six daily east-west transects were performed in the northern part of the lake twice a month in August and September 2009 and once in October 2009. Three points were sampled for each transect. A fluoroprobe (BBE, Moldaenke, Germany) specially calibrated to estimate *P. rubescens* biomass was used at each sampling point [23] and the thermocline depth was followed in parallel. At each sampling point, 2 L water samples were taken at three depths (6 m, 12 m and the depth of *P. rubescens* maximum density) for MC analysis. The water samples were passed through a filter (pore size 1 µm, Nucleopore, Whatman) and stored at −20°C until analysis.

The schooling of perch during the day [24], [25] and their position in the water column were studied using hydroacoustic methods [26] as described previously [27].

The intake of cyanobacterial toxins may result from the consumption of intoxicated prey [16], [28]. Prey items were identified by stomach content analysis. For this purpose, perch were sampled at night using a pelagic trawl [29] and stocked at 4°C until laboratory analysis. On the date of maximal concentrations of *P. rubescens* (27 August), zooplankton were sampled by several vertical tows at depths of 0 to 50 m with a 200 µm-mesh conical net and kept alive in cooler boxes until sorting through air bubbling techniques [30]. Then, Cladocerans were picked up on the surface of the water bubbled and immediately deep-frozen in liquid nitrogen until laboratory analysis.

### Zooplankton and fish sample preparation

In laboratory, Cladocerans samples were defrosted and *Daphnia* were then sorted out under a binocular microscope, thoroughly rinsed with ultrapure water to remove all attached *P. rubescens* filaments and then extracted for MCs analyses.

YOY perch were weighed, measured and dissected. Individual gut contents were observed under a microscope (magnification 40X). Ingested prey items were identified and counted at the genus level. Dorsal muscle and liver were sampled for each fish (n = 27) and preserved in formalin for histological studies. For MC analysis at each sampling date, the individual organ samples were pooled to provide 3 liver samples and 3 muscle samples.

### Laboratory experimental design

Experiments were performed in triplicate using YOY perch raised from eggs collected in Lake Geneva (France/Switzerland) and reared in tanks (research unit AFPA, Nancy University, France).

The perch were acclimatised for 1 week prior to experiments in outdoor tanks (480 L) supplied with water from Lake Geneva that was free from toxic cyanobacteria and was pumped continuously from a depth of 36 m. For each replicate, 440 fish (mean weight 3.36±0.24 g) were starved for 24 hours prior to treatment and were then distributed in cylindrical 20-L cages (n = 22 fish per cage) that were placed in clean large tanks (1140 L) filled with lake water at 20°C. The fish were anesthetised in water containing 300 µL propiscin per L (0.2%). For each replicate, 110 fish were individually force-fed through a catheter connected to a Hamilton syringe with 10 µL of a 5 mg.L^−1^ MC-LR solution (i.e., approximately 0.05 µg MC-LR per fish) or with 10 µL of a 50 mg.L^−1^ MC-LR solution (i.e., approximately 0.5 µg MC-LR per fish). MC-LR (Gentaure Corporation, France) was dissolved in water containing 0.04% phenol red to ensure that the force-feeding was successful. The MC concentrations of the force-fed solutions were confirmed by HPLC-PDA. The chromatographic resolution of MC-LR was achieved using the following mixture of MilliQ-water –0.3% (v/v) trifluoroacetic acid (A) and acetonitrile (B) as the mobile phase, in an elution gradient run on a chromatographic system (Waters) equipped with Waters 600 pump controller, a PDA 996 photodiode-array detector, a C18 PHENOMENEX Luna 5 mm (4.6 mm×250 mm) column and Waters3.2 millennium chromatography manager software. The flow rate was 1 ml/min and MC-LR peak was identified on the basis of their characteristic absorption spectra (maximum absorption at 238 nm) and its retention time [27]. As a placebo treatment, 110 fish were force-fed with 10 µl of a 0.04% neutral solution of phenol red to evaluate the effect of phenol red on the measured endpoints. The control treatment consisted of 110 fish that were not force-fed. During each experiment, the fish were not fed and were maintained at a water temperature of 20°C, a dissolved oxygen concentration of 11.7±0.2 mg.L^−1^, a pH of 8.1±0.3 and a natural light/dark cycle of 16/8 h.

At 6, 12, 24, 48 and 96 h after the start of each experiment, 10 fish per treatment were killed, weighed, measured and dissected. Muscle tissues were sampled, pooled and stored at −80°C until analysis. A liver sample from each fish was divided into two equal parts to provide two pools (one for MC analysis and another for the measurement of enzyme activity) and stored at −80°C until analysis. For each treatment, blood from the caudal vein was collected in a heparinised syringe. The blood samples were diluted 100-fold in a cryopreservative buffer (250 mM sucrose, 40 mM trisodium citrate, 5% dimethyl sulphoxide (DMSO) adjusted to pH 7.6 with 1 M citric acid), deep-frozen in liquid nitrogen and stored at −80°C processing. For histological analysis, 2 fish per treatment were dissected and liver and intestine samples were fixed immediately in formalin solution.

Environmentally relevant concentrations of 0.05 and 0.5 µg MC per fish (corresponding to 16.6 and 166 µg/kg body weight, respectively) were chosen for the experiments based on our previous observation that MC concentrations in zooplankton in Lake Bourget range from 0.07 to 3.5 µg/g dry weight (DW) when *P. rubescens* is present (unpublished data). We found that MC concentrations in cladocerans under experimental conditions reached 1.1 µg/g fresh weight (FW) for organisms fed with cyanobacteria concentrations equivalent to those measured during the summer blooms in Lake Bourget (unpublished data). Karås and Thoresson [31] reported a maximum daily food consumption of approximately 15% of fresh body weight for YOY perch. We therefore estimate that fish weighing approximately 3 g could ingest, *via* zooplankton, 0.05 µg MCs per day at the beginning of the bloom and 0.5 µg MCs per day at the height of the bloom.

### MC extraction and analysis

For MC analysis of *P. rubescens*, the extraction and measurement procedures described by Sotton et al. [27] were used. Zooplankton and YOY perch samples were ground, homogenised in 100% methanol, sonicated in an ultrasonic bath (Elma Sonic) for 5 min and then centrifuged (Beckman AVANTI J30 I) (24000 g, 45 min, −5°C) to precipitate the co-extracted proteins [32]. This extraction procedure was repeated twice. The supernatants from each extraction were collected with a 5-mL syringe and washed with hexane. The methanol layers were filtered through a syringe filter (pore size 0.2 µm; PTFE, Whatman), pooled and evaporated to dryness at 40°C with a SpeedVac device (SpeedVac Plus SC110A, Savant). Each dry extract was dissolved in an adequate volume of Milli-Q water; this volume varied depending on the sample. ELISA analyses were performed using a Microcystins (Adda-specific) Plate Kit (Abraxis LLC) [33], [34] and absorbances at a wavelength of 450 nm were determined using a microplate reader (Dynex MRX II, Dynex Technologies, Inc., USA). The results are presented as ng MC-LR equivalents per g fresh weight (FW). This method for the quantification of free MC concentrations was chosen because of its sensitivity and specificity. However, the method does not allow the discrimination of pure MC-LR from its metabolites and may have led to the overestimation of pure MC-LR concentrations in our samples [35], [36].

### GST activity measurement

Liver samples from experimental YOY perch were homogenised in cold phosphate buffered saline (PBS) and centrifuged at 10000×*g* for 15 min at 4°C. The supernatant was collected, and protein content was measured by spectrophotometry at 595 nm (Varian Cary 50 Spectrophotometer, Agilent Technologies, USA) according to the method of Bradford (1976). GST activity was determined by fluorometry using a GST Fluorometric Assay Kit (Biovision, USA) and a microplate reader (Fluoroskan Ascent FL, Thermo Fisher Scientific, France) at excitation wavelength 380 nm and emission wavelength 460 nm. The results are reported as U per mg total protein content, where U equals the quantity of enzyme that catalyses the transformation of 1 µmol substrate per minute.

### DNA damage assessment

DNA damage was measured in erythrocytes by the alkaline Comet assay using a slight modification of the procedure of Singh et al. [37]. The blood cells were thawed rapidly just before the assay, the cell density was adjusted to 10^3^ cells.µL^−1^ cold PBS, and the cell viability was checked by the trypan blue exclusion method [38]. The cell viability was found to be >90% in all experiments. Microscope slides were immersed in melted normal agarose prepared in PBS (final concentration 0.8%) and dried overnight at room temperature. A 50-µL aliquot of 1% low melting point agarose in PBS mixed with an equal volume of cell suspension was spread on the slide and a coverslip was placed. The agarose was solidified on an ice-chilled plate, a second layer consisting of 90 µL of 0.5% low melting point agarose was spread on the first layer, a coverslip was placed, and the agarose was again solidified by chilling. The subsequent steps were performed under a dim red light to avoid artificial DNA damage. The cells were treated with a lysing solution (2.5 M NaCl, 0.1 M EDTA, 10 mM Tris, pH 10, 1% (v/v) Triton X-100) and 10% (v/v) DMSO for 1 h at 4°C, and the DNA was allowed to unwind for 40 min in an electrophoresis buffer (0.3 M NaOH, 1 mM EDTA, pH >13). Electrophoresis was run for 24 min at 20V and 300 mA (0.6 V/cm). The slides were neutralised by Tris buffer (0.4 M Tris, pH 7.5) and dried for 15 min in absolute ethanol. The DNA was stained with 0.05 mM ethidium bromide and scored using an Axioskop epifluorescence microscope (Zeiss, Germany) and the Comet assay IV image analysis system (Perceptive Instruments Ltd., UK). Randomly selected cells from two replicate slides (50 cells per slide) were analysed. Of the Comet parameters, the tail intensity (percentage of tail DNA) is the most relevant [39]. Comet figures that showed an extremely prominent tail and that lacked a clearly identifiable head were excluded from the analysis because such figures may originate from necrotic or apoptotic processes [40].

### Histopathological analysis

Liver and intestine samples were fixed in buffered 10% formaldehyde solution for 48 hours, placed in a 70% ethanol solution, dehydrated in successive ethanol baths, embedded into blocks of paraffin wax and cut into 3.5-µm sections. The slides were rehydrated in successive 100% and 95% ethanol baths and stained with hematoxylin-eosin-saffron (HES) by a standard procedure. Periodic acid-Schiff (PAS) staining was performed to reveal the glycogen content of liver cells. Staining was performed according to standard histological procedures [41].

### Statistical analysis

Comparisons of fish survival rates between treatments at each sampling time were performed using the nonparametric Mann-Whitney test at *p*<0.05. MC-LR accumulations at different sampling times were compared in liver and muscle and for each dose using the nonparametric Kruskal-Wallis test followed by multiple pairwise comparisons using the Conover-Iman procedure. Comparisons of MC-LR accumulations between treatments at each sampling time were also performed using the nonparametric Mann-Whitney test. Differences were considered significant at the *p*<0.05 level for all statistical analyses. Comparisons of GST activity between treatments at each sampling time were performed using the nonparametric Mann-Whitney test at *p*<0.05. Because the distribution of DNA damage measured by the Comet assay does not follow a Gaussian distribution, both the nonparametric Kruskal-Wallis and Mann-Whitney tests were used for data analysis. All statistical analyses were performed using the XLSTAT 2011 software program.

## Results

### *In situ* observations

**Figure 1:**
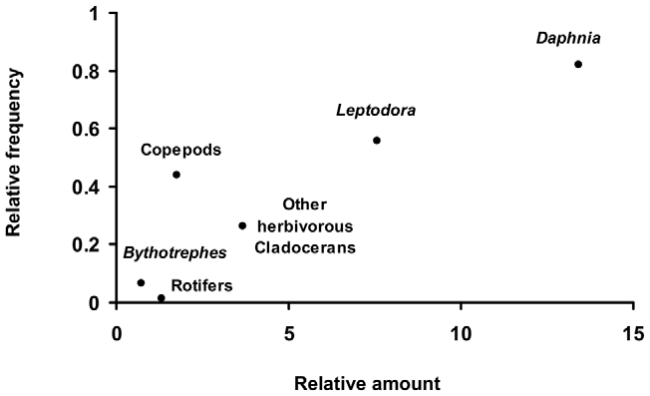
Average prey preference of YOY perch during the studied period. Average prey specific abundance observed in the stomach content (X axis) plotted against the average frequency of occurrence of the prey in the diet (Y axis).

The YOY perch preyed on various zooplankton taxa during the study period. Their diet consisted mainly of *Daphnia* and *Leptodora*. Copepods and other herbivorous cladocerans (*Bosmina, Diaphanosoma*) were also present in the stomach contents but had a lower abundance and frequency. *Bythotrephes* and rotifers were found occasionally and had low abundance (Fig. 1).

**Figure 2:**
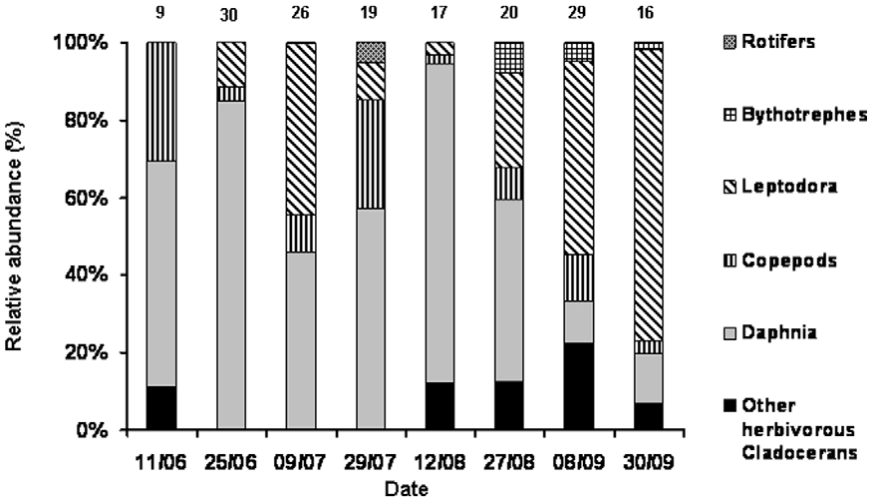
Temporal changes of the relative abundance of the zooplankton prey taxa observed in stomach contents. The number of analysed stomach contents is indicated above the bars.

The relative abundance of these prey taxa fluctuated during the summer. At the beginning of August, *Daphnia* was the dominant taxon and comprised 80% of the YOY diet (Fig. 2); this percentage declined steadily until it reached a low value of 10%. Meanwhile, the percentage of *Leptodora* in the diet increased steadily and reached a maximum of 75% in September. The diet was dominated by herbivorous taxa in August but by carnivorous taxa in September.

**Figure 3:**
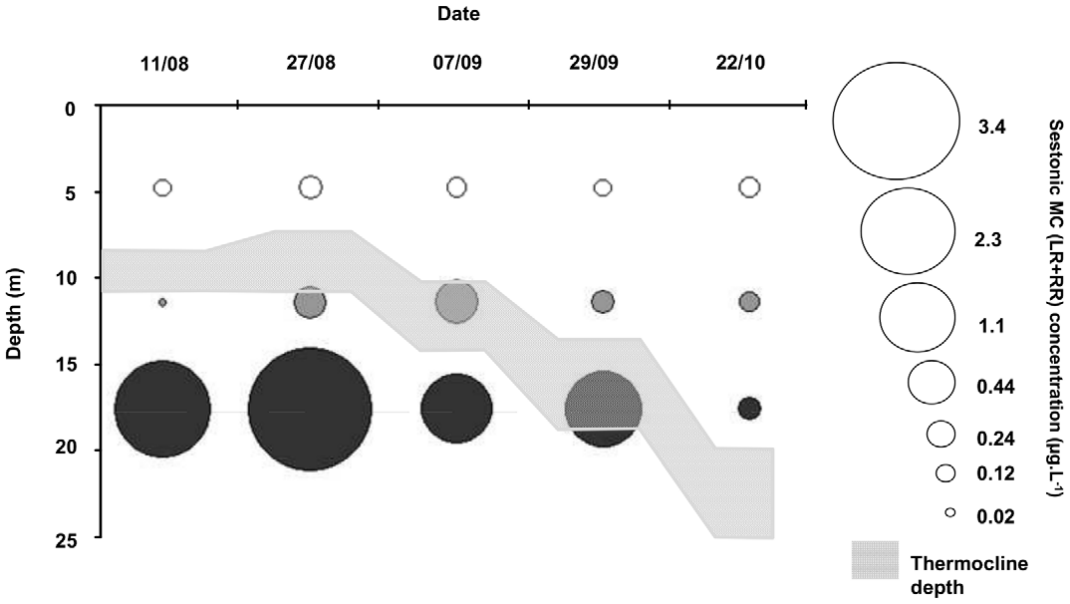
Sestonic microcystin (LR+RR) concentrations at 6 m (white), 12 m (grey) and at the *P. rubsencens* peak depth (black) in Lake Bourget during summer 2009. To facilitate the figure reading, the mean of peak depths was calculated for each date.

The YOY perch were located exclusively in the epilimnion during the entire sampling season. At this depth, the MC concentration remained ≤0.12 µg.L^−1^ (Fig. 3). The highest MC concentrations were found between 15 and 20 m, which was the depth of maximal *P. rubescens* abundance as detected by the BBE probe. During the summer, the maximal MC concentrations were found below the thermocline. At the depth of maximal *P. rubescens* abundance, MC concentrations were highest in August, with average concentrations that ranged from 2.3 to 3.4 µg.L^−1^ (Fig. 3).

**Figure 4:**
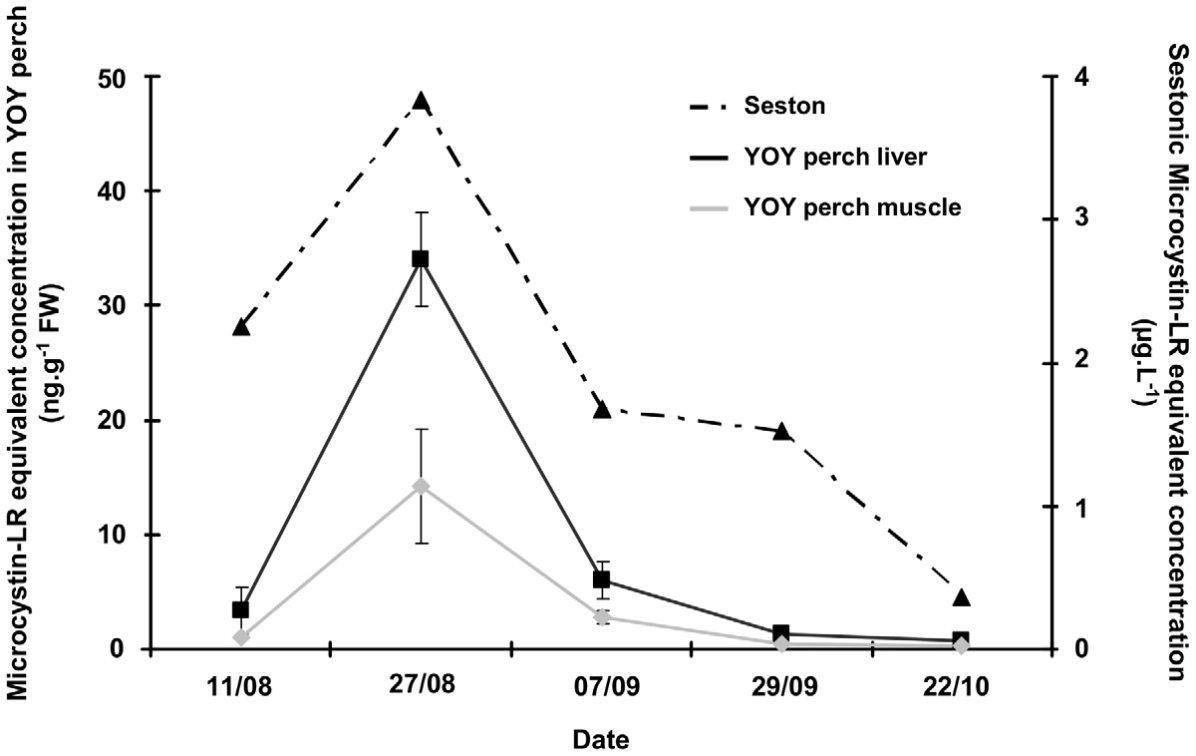
Microcystin concentrations in liver and muscle of YOY perch and in seston during summer 2009 in Lake Bourget. Sestonic MC-LR equivalent concentrations are expressed as µg.L^−1^ and correspond to the sum of average concentrations found at the three depths sampled (6 m, 12 m, *P. rubescens* peak depth). MC-LR equivalent concentrations measured in organs are expressed as ng.g^−1^ fresh weight.

Although no spatial correlation between perch and *P. rubescens* abundance was observed, MC was detected in perch liver and muscle samples from August to October. The maximal MC concentrations (34 ng.g^−1^ FW in liver; 14 ng.g^−1^ FW in muscle) were detected on the date (27 August 2009) that the maximal sestonic MC-LR equivalent concentrations were measured in the water column. Subsequently, a significant (*p*<0.05) decrease in MC concentration was observed in both organs. Significant positive correlations were observed between the MC concentration in seston and those in liver (R^2^ = 0.7221) and in muscle (R^2^ = 0.6369) samples from YOY perch. Regardless of the date, MC concentrations were higher in liver than in muscle (Fig. 4). On the 27 August, when the MC concentrations in *P. rubescens* and seston in the water column were maximal (Fig. 4), the MC-LR equivalent concentration in *Daphnia* was 422 ng.g^−1^ FW.

No histological alterations were observed for any sampling date or any of the organs studied.

### Experimental study

#### Microcystin accumulation

The mortality rate never exceeded 10% in the experimental groups. There were no significant differences in mortality between treatments regardless of the sampling time (data not shown).

**Figure 5:**
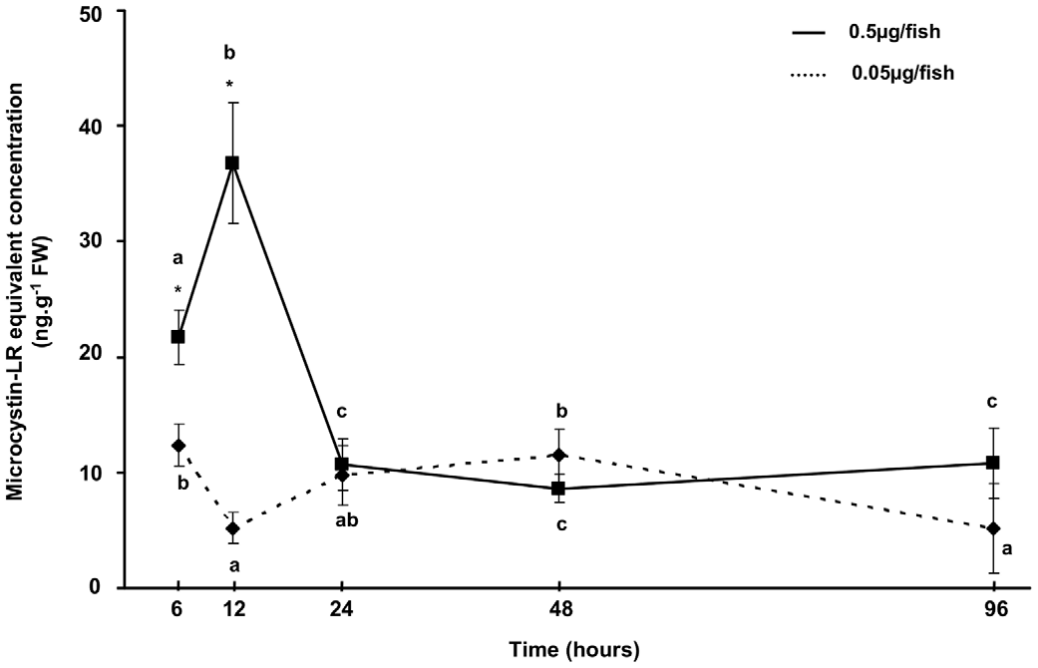
MC-LR accumulation in liver of YOY perch exposed to two relevant doses, 0.5 µg/fish and 0.05 µg/fish. * corresponds to a significant difference observed between microcystin treatments (Mann-Whitney, *p*<0.05). MC-LR equivalent concentrations are expressed as ng.g^−1^ fresh weight. Different letters correspond to a significant difference between sampling times (Mann-Whitney, *p*<0.05).

**Figure 6:**
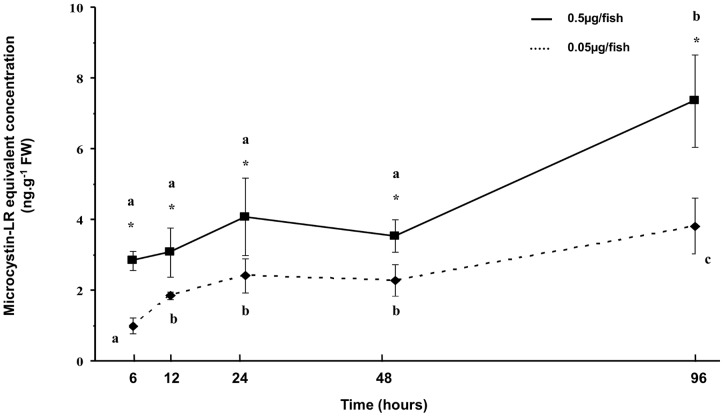
MC-LR accumulation in muscle of YOY perch exposed to two relevant doses, 0.5 µg/fish and 0.05 µg/fish. * corresponds to a significant difference observed between microcystin treatments (Mann-Whitney, *p*<0.05). MC-LR equivalent concentrations are expressed as ng.g^−1^ fresh weight. Different letters correspond to a significant difference between sampling times (Mann-Whitney, *p*<0.05).

Regardless of the experimental MC concentration or the sampling time, no MC was detected in either the control or placebo groups. For both of the MC-LR treatments, the toxin was detected in the liver and muscle samples of perch from the first sampling time (6 h) until the end of the experiment (Figs. 5 and 6). At 6 h, the MC concentrations detected in both organs were dose-dependent, and the concentrations in the liver were higher than those in muscle. In the liver, for the higher experimental MC-LR dose, a significant increase (*p*<0.05) in MC-LR concentration was detected at 12 h. The concentration was 37 ng.g^−1^ FW at 12 h and then fell rapidly to reach a significantly (*p*<0.05) lower value at 24 h (Fig. 5). In the liver, for the lower experimental MC-LR dose, the MC-LR concentration was significantly (*p*<0.05) lower at 12 h, increased gradually until 48 h when it reached its maximal value and then declined to reach a significantly (*p*<0.05) lower value at 96 h (Fig. 5). In the muscle, significant increases in toxin concentration were observed for all experimental MC-LR doses (Fig. 6). At the end of the experiment, The MC-LR concentration was approximately 7 ng.g^−1^ FW for the higher experimental dose and 4 ng.g^−1^ FW for the lower experimental dose (Fig. 6).

#### GST activity

**Figure 7:**
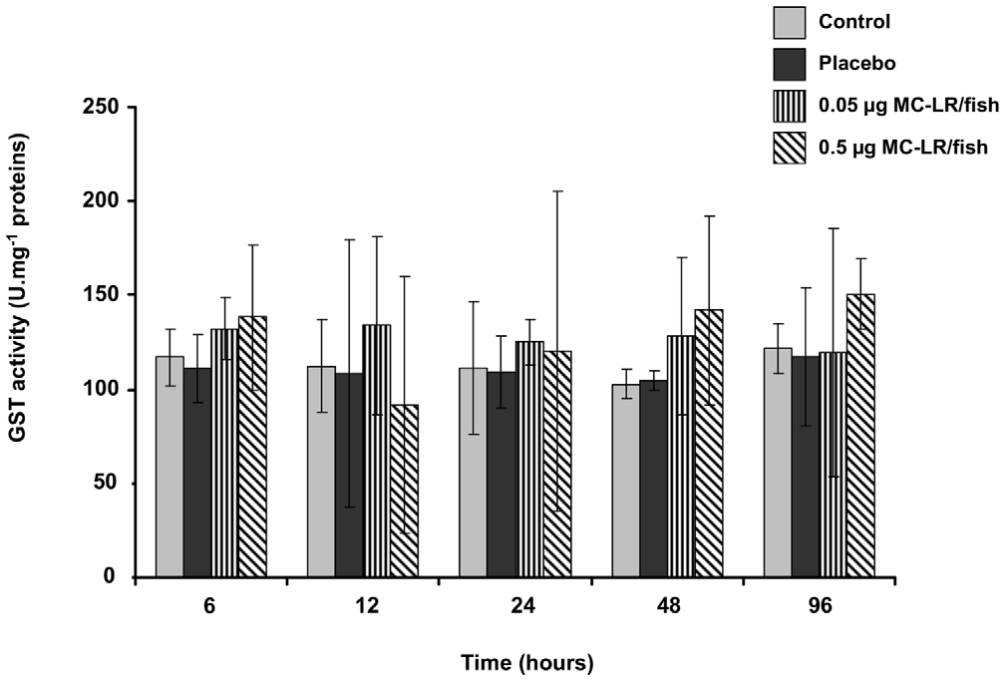
Glutathion S-transferase (GST) activity in liver of YOY perch force-fed with two doses of MC-LR (0.05 µg and 0.5 µg/fish). GST activity is expressed as U.mg^−1^ proteins.

Regardless of the sampling time, no significant difference in GST activity was found in the control vs. placebo groups or in the control vs. experimental groups (Fig. 7). There was high variability among the replicates.

#### Histology

**Figure 8:**
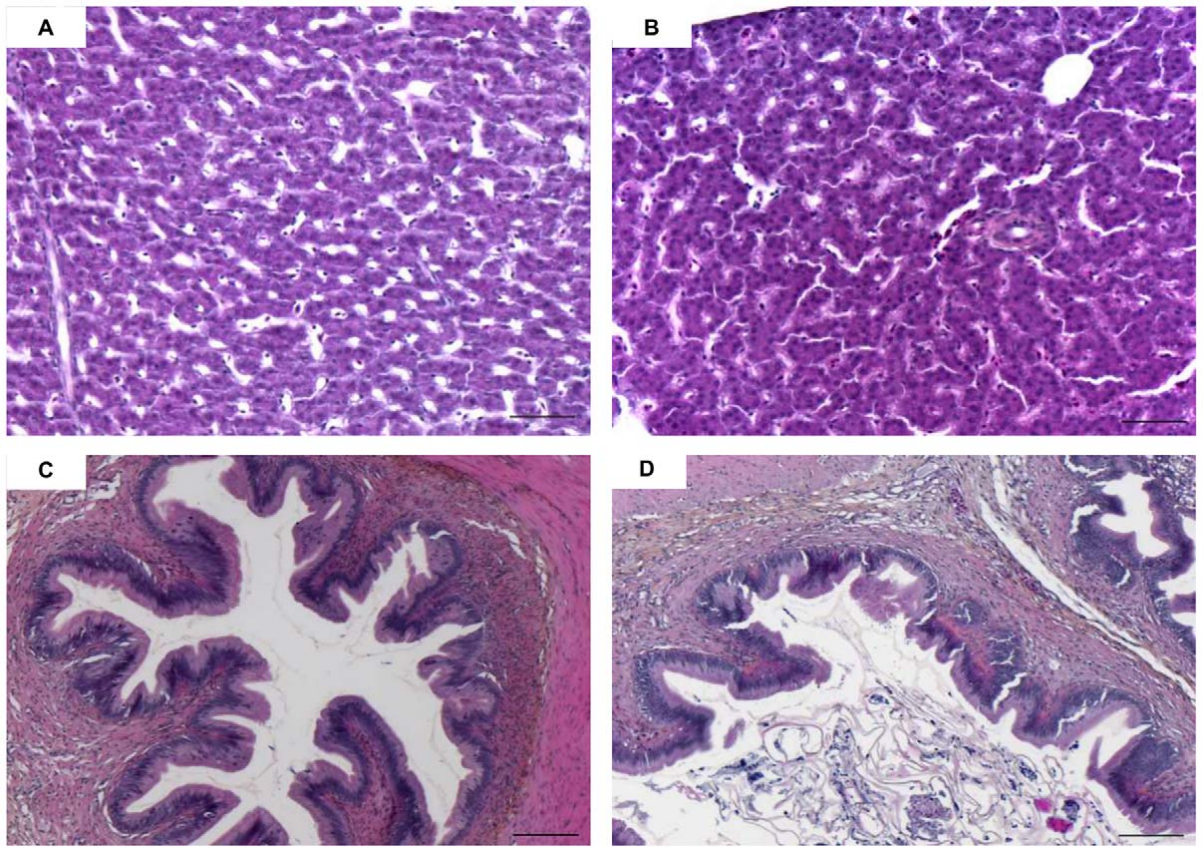
Light microscopy transverse sections of liver (A–B) and intestine (C–D) of control (A–C) and treated (0.5 µg of MC-LR) (B–D) YOY perch, stained with hematoxylin–eosin–saffron. Scale bar: 50 µm for liver and 100 µm for intestine.

**Figure 9:**
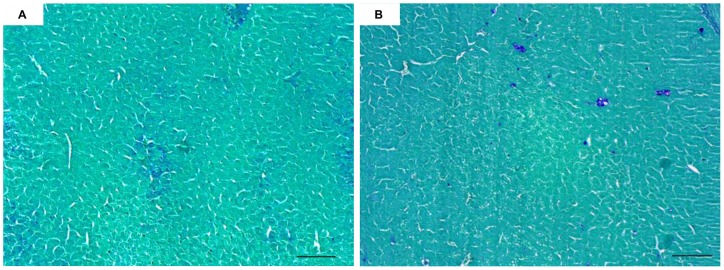
Light microscopy transverse sections of liver control (A) and treated (0.5 µg of MC-LR) (B) YOY perch, stained with periodic acid-Schiff. Scale bar: 100 µm.

Regardless of the sampling time or the organ studied (liver vs. intestine), no significant histological differences were observed among the placebo, control and experimental groups (treated with 0.5 or 0.05 µg MC-LR/fish) (Fig. 8). Regardless of the sampling time, PAS staining did not reveal any differences in glycogen storage or consumption in the livers of the control vs. experimental groups (Fig. 9).

#### DNA damage

**Figure 10:**
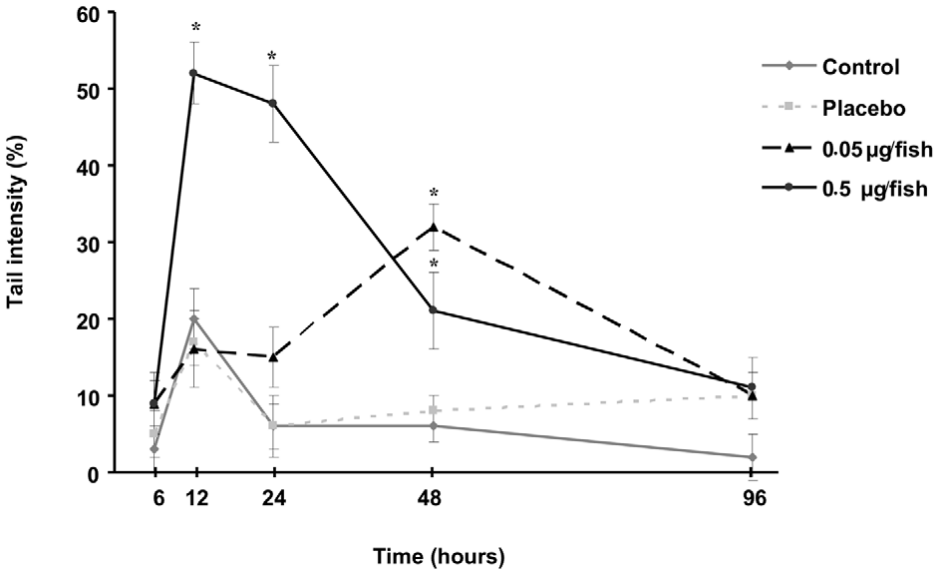
DNA damage in YOY perch erythrocytes measured through the comet essay. Star indicates significant differences between the different treatments and control group values (Mann-Whitney, p-value<0.05).

Significant DNA damage, expressed as % tail intensity, was observed in the erythrocytes of the experimental group (Fig. 10). Basal DNA damage in the placebo and control groups remained in the range of 3 to 20% tail intensity throughout the study. A 3-fold increase in DNA damage was observed 12 hours after the initial force-feeding for the higher MC-LR dose. A clear trend of decreasing DNA damage with time was observed in the group treated with 0.5 µg MC-LR, although DNA damage after 48 h was still significantly greater in comparison with the control group. The induction of DNA damage in the group that received the lower MC-LR dose was delayed relative to the group that received the higher dose in that the damage was significantly increased (5-fold) in comparison with the control group only after 48 h. Regardless of the MC-LR dose, none of the experimental groups displayed evidence of genotoxicity greater than that of the control group after 96 h.

## Discussion

Two points regarding the MC concentrations measured in our study must be discussed. Firstly, only free MCs were measured. MCs undergo a two-step interaction with phosphatase proteins 1 and 2A [42]: (i) a rapid and reversible binding with the phosphatases and (ii) a covalent linkage between the N-methyldehydroalanine residue (Mdha) of the MCs and a nucleophilic site on the phosphatases, leading to irreversible inactivation [43], [44]. To measure the total MC concentration, the free and the covalently bound MCs must be extracted from the sample by two different methods [45]. It has been estimated that 38–99% of MCs [28], [46], [47] may be covalently linked to phosphatases and thereby unextractable by the organic solvents that are used for free MCs. The measurement of only free MCs may therefore lead to an underestimation of total MC concentrations in samples. Secondly, the free MC concentrations measured in liver and muscle samples from YOY perch are most likely greater than the real concentrations of pure free MC-LR that are present in these two organs. Our ELISA method, which targets molecules that possess an unusual β-amino acid (ADDA; 4*E*, 6*E* 3-amino-9-methoxy-2, 6, 8-trimethyl-10-phenyldeca-4, 6-dienoic acid), quantifies all molecules that possess ADDA as pure MCs but also so quantifies the detoxification products of MCs, which are less toxic [35]. Our method may therefore lead to an overestimation of free MC levels in YOY perch; i.e., the toxin values presented here may underestimate the total MC concentrations in samples as a result of the extraction method that targets only free MCs while the pure MC-LR concentrations of samples may be overestimated as a result of the ELISA measurements that quantify both pure MCs and their metabolites.

Previous studies have revealed differences between fish species in terms of their sensitivity to cyanobacterial toxins [15]. The number of feeding experiments on fish using MCs or cyanobacteria has greatly increased in recent years. However, few studies have focused on fish species endemic to the peri-alpine lakes in which *P. rubescens* blooms have occurred repeatedly during the past 10 years [17], [48], [49]. In particular, there have been no studies on the Eurasian perch, which is important both commercially and for sport fishing, is widespread throughout Europe and Asia and has been successfully introduced in other continents.

The mortality rates for YOY perch observed following force-feeding with environmentally relevant MC concentrations was low in the present study. Fish weighing 3 g were treated with 0.5 µg and 0.05 µg of MC-LR per fish; these doses correspond to approximately 166 µg/kg and 16.6 µg/kg body weight, respectively. The MC-LR concentrations used were well below the median lethal concentration values (LC50) obtained after injection of 550 µg MC-LR/kg body weight for common carp (*Cyprinus carpio*) and rainbow trout (*Oncorhynchus mykiss*) [50], [51]. This fact may explain the low mortality rates observed in our study. MC-LR was administered by force-feeding in the present study in contrast to the injection method using in the carp and trout studies. The route of exposure is presumed to be an important factor affecting toxicity. Tencalla et al. [51] demonstrated a higher toxicity of MC-LR administered by injection than by force-feeding in rainbow trout.

During the present study, MC-LR equivalent concentrations were detected in liver and muscle from 6 hours until 96 hours after treatment. In both of these organs, a rapid accumulation was observed for both the high and low MC-LR doses. In muscle, the MC-LR equivalent concentration increased for both doses in all of the experiments, and the highest concentrations found for the higher dose. In liver, the maximal MC-LR equivalent concentration was detected after 12 hours for the higher MC-LR dose and 6 hours for the lower dose. Following the peak, a decline in the MC-LR equivalent concentration was observed for both doses. These findings suggest that MC-LR may have been bound to PPase and thus undetected by our measurement method (ELISA) targeting only free MCs or excreted from the liver during the initial hours of the experiments; such processes may also occur in the field and influence the measurements. Similar results were observed previously on fish force-fed with toxic cyanobacteria [14], [52]. Before its excretion, MC-LR undergoes a complex detoxification process that involves various enzymes of which the most studied are the glutathione S-transferases (GSTs) which conjugate MCs to GSH [7]. In the present study, no significant difference in GST activity was observed between the control group and the experimental groups that were force-fed with MC-LR. This finding suggests that MC-LR ingested by YOY perch *via* zooplankton consumption does not induce an increase in the GST activity, which is consistent with other study [53], [54]. However in our study, MC-LR concentrations in the livers of YOY perch declined even though there was no change in GST activity. This observation could suggest that the basal level of GST activity in YOY perch was sufficient to detoxify the MCs concentrations that were administered in our fish. Furthermore, it could not be exclude the possible existence of another important MC-LR detoxification pathway involving conjugation of the toxin to molecules containing cystein residues (e.g. polypeptides, PPases) [55].

In the present study, no histological alterations were observed in the experimental groups for any of the organs studied. This finding is not surprising in view of other reports on this topic. Most histological alterations have been observed following immersion, intraperitoneal injection or even force-feeding but with acute doses of MCs [10], [56]. The liver, kidneys, gills and intestine are the organs most affected histologically by exposure to MCs. Histological alterations have been observed frequently following exposure to high MC concentrations or to a high biomass of cyanobacteria [15]. Thus, in both *in situ* and experimental studies, MC-LR concentrations are typically too low to induce histological damage in YOY perch. Differences in the administration routes of toxins and in the sensitivity of various fish species to cyanobacterial toxins are additional factors that may account for the differences in results among studies [15].

A transient increase in DNA damage was observed in perch erythrocytes following treatment with MC-LR, after 12 h and 48 h for the higher and the lower MC-LR dose, respectively. Reactive oxygen species are known to be produced following the exposure of fish to MCs [57], [58]. This can partially explain the genotoxicity observed after perch exposure to MC-LR. The possibility cannot be ruled out that the DNA damage observed in perch erythrocytes arises from other pathways such as the production of instable DNA adducts that could be revealed through the comet assay. However, such underlying mechanisms of toxicity remain to be confirmed in fish. Regardless of the MC-LR dose, DNA damage levels returned to those of the control group 4 days after treatment. Such decreases in DNA damage may be explained by DNA repair and/or erythrocyte turnover; it is not possible to take into account the formation of apoptotic erythrocytes during Comet assay image analysis because of the huge number of DNA strand breaks in apoptotic cells [59]. Genotoxic stress was thus observed in perch exposed to environmentally relevant MC-LR doses in terms of the toxin concentration ranges analysed in seston. YOY perch may therefore suffer drastic effects from the major algal blooms observed in peri-alpine lakes. This idea is consistent with the results of a previous study of another lake fish species, the European whitefish (*Coregonus lavaretus*) [60].

*P. rubescens*, a MC-producing cyanobacterium, is found frequently in the metalimnion of peri-alpine lakes during the summer [17], [18], [33]. The development of *P. rubescens* in this water layer is facilitated by various specialised features such as gas vesicles that adjust the position of the organism in the water column in response to the vertical gradient of irradiance [61], photosynthetic pigments rich in phycoerythrin protein that absorb the entire spectrum of photosynthetic active radiation (PAR) [62] and the excretion of alkaline phosphatases that utilise the organic forms of phosphorus when inorganic phosphate concentrations are low [62]. In the present study, high biomasses of toxic *P. rubescens* were observed in the metalimnion during the summer. However, data concerning the interactions of *P. rubescens* and MCs with fish species in Lake Bourget are still scarce.

In this study, schools of YOY perch were observed in the epilimnion of the pelagic area above the *P. rubescens* blooms. This vertical distribution is consistent with observations in other studies [25], [29], [63]. YOY perch are present in the epilimnion because of their thermal preferendum for the 16–26°C range [64]. Thus, YOY perch are spatially separated and have no direct contact with *P. rubescens* and their toxins. However, MCs were detected in the liver and muscles of YOY perch, and the maximal MC concentrations for both organs were found in August when the maximal MC concentrations in seston were also observed. MCs accumulation in perch has already reported in bloom conditions by several authors before [28], [65], [66]. The MCs found in the fish are derived principally from their food sources. *Daphnia* are a major component of the perch diet and are known to be contaminated by MCs; i.e., *Daphnia* ingest filamentous cyanobacteria such as *P. rubescens* and consequently accumulate MCs [67], [68]. In Lake Bourget, *Daphnia* therefore appear to be a major vector of MCs to zooplanktivorous fish. The diel vertical migration (DVM) characteristic of *Daphnia* species [69] is an important factor leading to the ingestion of *P. rubescens* filaments. While passing through the metalimnion, *Daphnia* ingest *P. rubescens* filaments, accumulate MCs and are subsequently eaten by YOY perch in the epilimnion. Thus, the YOY perch in the lake are chronically exposed to MC *via* their food sources leading to contamination of fish by MCs. However, our experiment has shown that perch are able to detoxify and excrete a portion of ingested MC and thereby limit their toxin concentration. Such detoxification and excretion processes are active during chronic MC exposure, as indicated by the absence of a continuous increase in MC concentration. However, they do not allow the reduction in MC concentration as observed in our experiments and a significant correlation between MC concentrations in the YOY perch and in seston was observed during the *in situ* study. Our experimental results suggested that the tested MC concentrations did not induce histological damage and our field observation showed that even with chronic exposure the MC concentrations recorded in Lake Bourget did not induce histological damage in YOY perch. Genotoxic impacts were not assessed *in situ* because of the complexity of measuring the effect of a targeted molecule on these parameters under *in situ* conditions. Other substances may well affect these parameters and thereby confound the real impact of MCs on perch. Our laboratory experiments under controlled conditions allowed us to assess the negative genotoxic impact (i.e., DNA damage) of MC-LR on YOY perch and to conclude that similar effects on YOY perch may occur during *P. rubescens* bloom conditions.

Finally, perch are highly commercialized for human consumption and YOY are used to be eaten fry and completely. As a consequence, the results of that study raise questions about the possible human threat linked to the consumption of wild perch. Concentrations in muscle of YOY perch raise 14.2 ng.g^−1^ fresh weight (FW) in August during the bloom of *P. rubescens*. The WHO has established recommendations [70] concerning the amount of cyanotoxin that an individual can consume per day for a lifetime, the microcystin total daily intake threshold (TDI) (0.04 µg of microcystin/kg of body weight/day). Thus, with concentrations found in our study in August, a man weighting 80 kg and consuming around 150 g of perch fillets (a typical restaurant portion) absorbs 2.13 µg of microcystin per day which is below the TDI (3.2 µg/day for a man weighting 80 kg). Consequently, the consumption of perch fillets coming from the Lake Bourget seems to be safety for people, even during severe *P.rubescens* bloom as it occurred in 2009 compared to previous years. However, as previously discussed, the methodological biases do not allow a true estimation of the MCY concentrations in the fish tissues analysed. A as consequence, our results clearly underline the human exposure to MCs through the consumption of fish caught during *P. rubescens* blooms but more studies are needed to estimate the real risk link to the consumption of that fish.

## Conclusion

During the *P. rubescens* bloom in Lake Bourget in 2009, YOY perch populations were located in the epilimnion because of their thermal preferendum. The YOY perch were therefore never in direct contact with the *P. rubescens* bloom, which was located in the metalimnion. However, the YOY perch were exposed to MCs through the consumption of *Daphnia*. The MC concentrations ingested by YOY perch in the lake during 2009 did not result in an accumulation of the toxin sufficient to be unsafely for the human consumption or to induce lethal histological damages in fish. Indeed, YOY perch are able to detoxified and excreted rapidly MCs to avoid most of their negative impacts. However, genotoxic damage could occur even at low concentrations of MCs ingested possibly leading long term negative impacts on perch populations.
